# False-Positive Maternal Serum Screens in the Second Trimester as Markers of Placentally Mediated Complications Later in Pregnancy: A Systematic Review and Meta-Analysis

**DOI:** 10.1155/2021/5566234

**Published:** 2021-06-04

**Authors:** Christy L. Pylypjuk, Joel Monarrez-Espino

**Affiliations:** ^1^Department Obstetrics, Gynecology and Reproductive Sciences, University of Manitoba, Winnipeg, Canada R3A 1R9; ^2^Children's Hospital Research Institute of Manitoba, University of Manitoba, Winnipeg, Canada R3E 3P4; ^3^Department of Epidemiology and Population Health, University of London (London School of Hygiene and Tropical Medicine), London WC1E 7HT, UK; ^4^Department of Health Research, Christus Muguerza Hospital Chihuahua - University of Monterrey, Chihuahua 31000, Mexico; ^5^Department of Global Public Health, Karolinska Institute, Stockholm SE-171 77, Sweden

## Abstract

**Background:**

Multiple-marker, maternal serum screening (MSS) has been the cornerstone of prenatal diagnosis since the 1980s. While combinations of these markers are used to predict fetal risk of Down syndrome and other genetic conditions, there is some evidence that individual markers may also predict nongenetic pregnancy complications, particularly those related to placental dysfunction. The objective of this meta-analysis was to investigate the utility of false-positive, second-trimester MSS for Down syndrome as a marker of placentally mediated complications amongst singleton pregnancies globally.

**Methods:**

Electronic searches of PubMed, Medline, Embase, CINAHL, Web of Science, Scopus, and grey literature to 2019 were performed to identify observational studies comparing risk of pregnancy complications amongst pregnancies with false-positive MSS versus controls. A random-effects model of pooled odds ratios by outcome of interest (stillbirth, preeclampsia, fetal growth restriction, and preterm birth) and subgrouped by type of MSS test (double-, triple-, and quadruple-marker MSS) was used.

**Results:**

16 studies enrolling 68515 pregnancies were included. There were increased odds of preeclampsia (OR 1.28, 95% CI 1.09-1.51) and stillbirth (OR 2.46, 95% CI 1.94-3.12) amongst pregnancies with false-positive MSS. There was no significant association with preterm birth or growth restriction.

**Conclusions:**

There is some evidence of an association between false-positive, second-trimester MSS for Down syndrome and increased odds of preeclampsia and stillbirth. Future large-scale prospective studies are still needed to best determine the predictive value of false-positive MSS as a marker of placentally mediated complications later in pregnancy and evaluate potential clinical interventions to reduce these risks.

## 1. Introduction

With the advent of cell-free fetal DNA for prenatal diagnosis of Down syndrome, prenatal screening technology is evolving quickly [1, 2]. Multiple-marker, combined maternal serum screening (MSS) has been available since the 1980s and was the mainstay of original prenatal screening programs for fetal aneuploidy [1–3]. As an optional test offered to pregnant women between 15 and 20 weeks of gestation, second-trimester MSS involves a blood draw to measure the levels of up to four serum markers: alpha-fetoprotein (AFP), human chorionic gonadotropin (hCG), unconjugated estriol (uE3), and dimeric inhibin-A (DIA) [1–4]. These markers are then used in combination to calculate the individual pregnancy risk of fetal Down syndrome [1–4]. But with a false-positive rate of ~5%, many pregnancies that screen positive for Down syndrome by MSS do not actually have the condition [3, 5]. While many centres have transitioned to first-trimester or combined first- and second-trimester screening tests, second-trimester MSS remains the only serum marker-based aneuploidy screening test available for women presenting late for prenatal care or for whom the recognition of pregnancy was delayed (later than the first trimester). Serum-based multiple-marker MSS performed in the second trimester also remains the main option for many publicly funded prenatal screening across the world and is the exclusive option for screening in our health region.

There is some evidence that the levels of individual serum markers assessed by MSS to determine risks of fetal aneuploidy may also be used to prognosticate other pregnancy complications resulting from impaired placentation [6]. As such, in the setting of a structurally normal fetus with normal genetic testing, these serum markers may instead reflect impaired placental implantation and functioning [6–9]. For instance, pregnancies complicated by abnormal AFP levels show an increased risk of placental vascular lesions and chorionic villitis and are associated with higher rates of fetal growth restriction [10–13]. High levels of hCG have been attributed to hypoxic placental cells and related pregnancy complications [11–14]. Fetal loss, growth restriction, hypertensive disorders of pregnancy, preterm birth, oligohydramnios, and abruption have all been described in the context of abnormal AFP, hCG, uE3, and DIA levels in pregnancy [10–20]. However, there remains no clear guidance in the literature about the exact magnitude of risk or about how such pregnancies with false-positive MSS results should be managed [6].

To date, there has been no quantitative synthesis specifically describing the utility of false-positive, second trimester MSS using the screening cut-off for Down syndrome as a marker of nongenetic pregnancy complications. A few reviews have attempted to summarize the risks associated with single serum marker aberrations or random combinations of markers, but due to the heterogeneity of these studies and their respective patient populations, the results are inconsistent [21–23]; variable cut-offs of individual serum markers or random combinations of markers also limit the clinical application of such fundings. Furthermore, many of the individual studies were small and underpowered to adequately assess risk for some of these rarer adverse pregnancy outcomes [9, 24–26]. Placentally mediated complications of pregnancy (preeclampsia, fetal growth restriction, preterm birth, and stillbirth) confer considerable risk for mothers and newborns worldwide. Hypertensive disorders of pregnancy, including preeclampsia, are the leading cause of direct obstetric deaths for women in high-income countries [27], and preterm birth is the leading cause of infant mortality worldwide [28]; the possibility of early prediction would afford the chance of earlier intervention and prevention strategies to improve outcomes and reduce associated morbidity.

The purpose of this review was to investigate the utility of false-positive, second-trimester MSS (using exclusively the screen-positive cut-off for Down syndrome) as a marker of placentally mediated complications later in pregnancy amongst singletons globally. The placental complications under consideration include preeclampsia, fetal growth restriction, preterm birth, and stillbirth. If there is an association between false-positive MSS results and complications, then this information would better inform healthcare providers about the risks and potential need for increased surveillance and possible intervention [29]. Conversely, if there is no association, patients can be reassured by a normal amniocentesis and spared the additional time and costs of increased surveillance. The results of this review also have the potential to inform future prenatal screening programs, particularly given the arrival of newer screening modalities for fetal aneuploidy which may or may not provide additional information about other pregnancy risks beyond genetic problems [1, 2].

## 2. Materials and Methods

This review was conducted in accordance with MOOSE guidelines. A study protocol was prepared a priori and reviewed as part of graduate coursework at the London School of Hygiene and Tropical Medicine (LSHTM) and subsequently published online (doi:10.17504/protocols.io.brngm5bw) [30, 31]. All observational studies assessing the association between multiple-marker, second-trimester MSS and adverse pregnancy outcomes were considered for this review. Only studies with both “exposure” and “control” groups were evaluated. The population of interest was women with singleton pregnancies undergoing multiple-marker, second-trimester MSS globally. In order to reduce potential confounding by pregnancy type, pregnancies of multiples were excluded. Four placentally mediated complications of pregnancy were chosen as the main outcomes of interest: (1) preeclampsia (blood pressure > 140/90 with proteinuria), (2) fetal growth restriction (weight less than the 10^th^ percentile at birth), (3) preterm birth (delivery prior to 37 weeks of gestation), and (4) fetal demise (fetal death in utero after 20 weeks of gestation). Diagnostic definitions of these outcomes were consistent with ICD-9 and ICD-10 diagnostic codes, except for fetal demise which was consistent with professional guidelines [6, 32–38]. Ethics approval for this review was sought from the Research Ethics Committee of the LSHTM at the University of London; however, this study was deemed exempt.

### 2.1. Search Strategy and Inclusion Criteria

Electronic searches of PubMed, Medline, Embase via Ovid, Web of Science, Cumulative Index to Nursing and Allied Health (CINAHL), and Scopus databases as well as hand searches of scientific meeting abstracts and other grey literature sources (ProQuest Dissertations and Google Scholar) to September 2019 were performed. The search strategy comprised of three general concepts: (1) “maternal serum screening,” (2) “false-positive results,” and (3) “adverse pregnancy outcomes.” Medical subject headings (or equivalent) with their related words and synonyms were used for all search concepts, and search strategies were then individualized to the specific databases mentioned previously. A sample search strategy is available in Supplementary Information Table S1.

To be considered eligible for inclusion, studies had to fulfill the following criteria: (i) singleton pregnancy, (ii) second-trimester, multiple-marker maternal serum screening test (double-, triple-, and/or quadruple-marker screens) with reported screen-positive cut-offs for Down syndrome, (iii) observational design with a suitable control group (i.e., cohort or case-control studies), (iv) report on ≥1 outcome(s) of interest, and (v) report at least one measure of association (either risk ratio or odds ratio) for the relationship between MSS test status and the development of adverse pregnancy outcomes, or report the raw number of events between groups so that a measure of association could be calculated by the reviewer. Exclusion criteria included the following: multiple gestations (twins or higher-order multiples), no screen-positive cut-off for Down syndrome reported (i.e., individual analyte levels utilized instead of a combined/integrated result), no control group, and/or non-English language publication.

### 2.2. Data Extraction and Quality Assessment

All citations were managed using RefWorks reference manager software (ProQuest LLC, Ann Arbor, MI, USA). The assessment was done in duplicate with discrepancies resolved by consensus. First, all titles were reviewed for possible relevance. Abstracts of relevant citations were screened for inclusion criteria, and potential studies were classified as follows: include, exclude, unclear, or duplicate. The full text of all reports classified as either “include” or “unclear” was then retrieved and reviewed for possible inclusion in this manuscript. Once the final roster of “included” papers was assembled, the full texts of all remaining studies were assessed using a standardized data collection form (Supplemental Information Figure S1). Data was then entered into a Microsoft Excel database (Excel v.14, Microsoft Corp., Redmond, WA, USA). Finally, a PRISMA flow diagram was constructed to illustrate the number of records reviewed [39].

Internal validity of included trials was assessed for bias using the Newcastle-Ottawa Screening tool (NOS) [40]. Studies classified as high risk of bias were excluded from the quantitative meta-analysis. Information on risk of bias for each study was used to explore possible sources of heterogeneity and in order to guide sensitivity analyses when relevant. The data from included studies was analyzed using Review Manager v5.3.5 (RevMan V.5.3.5, The Nordic Cochrane Centre, The Cochrane Collaboration, Copenhagen, Denmark).

### 2.3. Statistical Analysis

The statistical analysis was performed using Comparative Meta-Analysis (CMA) v2.0 software to allow for input of multiple different data types. A random-effects model was chosen to calculate the pooled odds ratios given that the background literature suggested there could be variability of the effect size amongst studies [41]. Pooled odds ratios were calculated for each outcome by subgroup of MSS testing (i.e., double-marker or triple-marker) and as a pooled, combined estimate overall. Whenever outcomes were reported by individual studies as number of events per group or as proportions, those values were transformed into odds ratios with 95% confidence intervals using the CMA statistical software when appropriate. Statistical heterogeneity of the data was determined using the *Q* statistic, Tau^2^, and *I*^2^ statistics. For the *I*^2^ test, uncertainty intervals were not planned a priori [41]. The suspicion of significant heterogeneity (*I*^2^ > 50%) was followed-up with further analysis when required to determine potential sources of the heterogeneity. Publication bias was assessed by the trim and fill, fail-safe N, and funnel plot methods (with imputed studies when less than 10 were found); further statistical evaluation of funnel plot asymmetry was performed using Egger's test [41]. Sensitivity analyses were planned a priori to evaluate the influence of maternal age and other tests of precision considered given the inherent risks of confounding and bias when combining. Strength of evidence was assessed using evidence of individual domains (risk of bias, inconsistency, indirectness, imprecision, publication bias, and other factors including upgrading) and GRADE methodology and ultimately classified as high, moderate, low, or very low [41, 42].

## 3. Results

### 3.1. Literature Search and Study Characteristics

**Figure 1:**
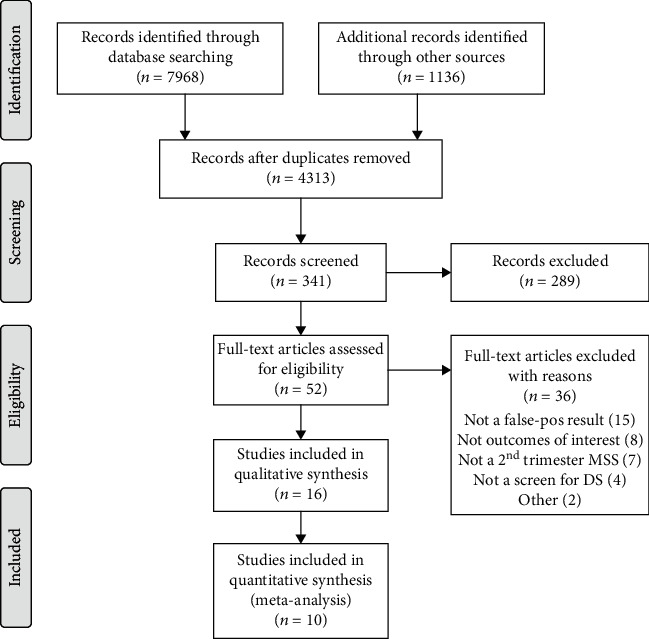
Selection of studies for inclusion in the meta-analysis.

Search of the literature identified 7968 publications from electronic databases and another 1136 citations from grey literature (Figure 1). After removal of duplicates, 4313 titles remained and were screened for possible relevance. Further screening of 341 abstracts yielded 52 applicable citations, of which full texts were reviewed. 36 citations were excluded because of the following reasons: did not report a composite false-positive maternal serum screen result (15), did not assess any of the four outcomes of interest (8), did not report a second-trimester serum screen (7), did not report a Down syndrome screening result (4), and 2 for other reasons. In the end, 16 studies met the criteria for inclusion in this systematic review (Figure 1).

The 16 studies published in September 2019 represent 68515 singleton pregnancies globally [24–26, 43–55]. Five studies examined second-trimester double-marker screening (AFP and hCG), 10 studies examined triple-marker screening (AFP, hCG, and uE3), and one study examined quadruple-marker screening (AFP, hCG, uE3, and DIA) (Table 1). Cut-offs for screen positivity were similar for all. Of studies eligible for inclusion, there was one case-control study and 15 cohort studies. Nine studies utilized an individually matched design, and one performed an adjusted analysis to account for differences between groups. 14 studies were based at university teaching hospitals while the remaining utilized large, regional, population-based genetic databases (Table 1). Geographically, there were 6 studies from North America, 4 studies from Europe, 3 from South Asia, and 2 from the Middle East.

The average maternal age ranged from 27 to 33.5 years old. Due to screening practices of the time, many studies only enrolled women under 35 because women older than 35 would have customarily been offered diagnostic amniocentesis testing directly instead of screening. Ascertainment of outcomes was obtained by a range of methods: database, hospital chart review, and/or interview (Table 1). In total, 7 studies reported on preeclampsia, 11 on fetal growth restriction (using less than 10^th^ percentile birth weight as the definition), 8 on preterm birth, and 9 reported on stillbirth. Of note, only one study used the WHO definition of stillbirth of fetal death in utero prior to 28 weeks of gestation, whereas the remaining 8 used the definition of fetal death after 20 completed weeks of gestation [32–35]. Baseline prevalence of the outcomes amongst controls varied widely from 0 to 11% for preeclampsia and 0 to 9% for fetal growth restriction and represented significant departures from the baseline rates expected in the general population [6, 34–38].

### 3.2. Risk of Bias

**Figure 2:**
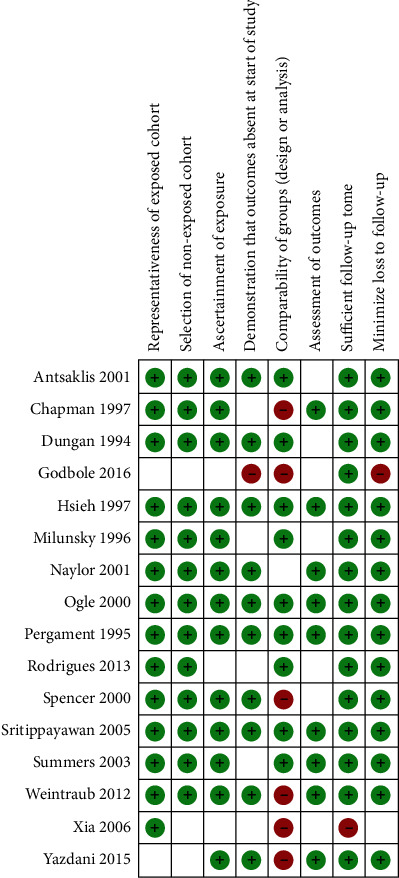
Risk of bias summary of review author's judgments for each risk of bias item for included studies.

**Figure 3:**
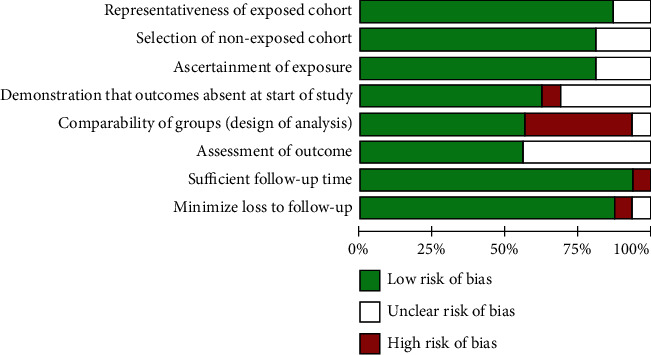
Risk of bias graph of review author's judgments for each risk of bias item (%) across studies.

The risk of bias amongst individual studies is represented graphically in Figure 2. The highest risk of bias came from the comparability of groups (~40%) and was mainly due to the lack of control for confounders/effect modifiers at either the design or analysis phase (Figure 3). Only 5% of risk across studies was due each to selection criteria (absence of outcomes at the start of study) and follow-up (sufficient follow-up time and loss to follow-up) biases. In total, 10 studies were considered of good or fair quality and included for quantitative meta-analysis [25, 26, 44–48, 50–52]. The single quadruple-marker study was excluded due to missing information about study methodology [55].

### 3.3. Quantitative Analysis of Outcomes and Subgroups

#### 3.3.1. Preeclampsia

**Figure 4:**
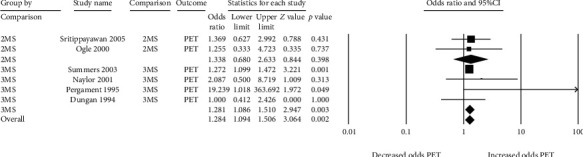
Forest plots of the relationship between false-positive MSS and preeclampsia (PET), subgrouped by double- (2MS) and triple-marker (3MS) test type.

Six studies assessed preeclampsia (Figure 4) [25, 26, 45, 47, 51, 52]. All studies except one showed that a false-positive MSS is associated with an increased risk of preeclampsia. The single study that did not show a positive association had a “neutral” result (i.e., odds ratio of 1) [47]. For the other studies, the odds of preeclampsia ranged from 1.27 to 19.24. This relationship seemed more robust when considering the results from the triple-marker tests as compared to the double-marker tests. The pooled odds ratio for the risk of preeclampsia amongst false-positive triple-marker screens was 1.28 (95% CI 1.09 to 1.51) versus 1.34 (95% CI 0.68 to 2.63) for double-marker screens (Figure 4). So while the magnitude of the association is somewhat variable, the direction of the association is consistent across studies. There was low heterogeneity amongst double-marker tests (*Q*‐df < 0, *I*^2^ 0) and moderate heterogeneity amongst triple-marker tests (*Q*-df 1.11), and 25.5% of this heterogeneity was assumed to be real (*I*^2^ 25.2%).

#### 3.3.2. Stillbirth

**Figure 5:**
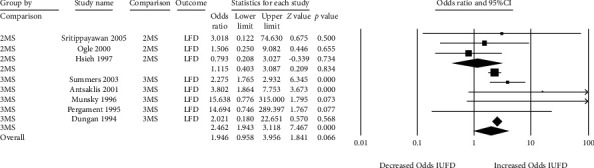
Forest plots of the relationship between false-positive MSS and stillbirth (IUFD), subgrouped by double- (2MS) and triple-marker (3MS) test type.

Concerning the association between false-positive MSS with stillbirth (also known as intrauterine fetal demise or “IUFD”), there were 8 studies [25, 26, 44, 45, 47, 48, 50, 52]. Seven studies showed increased odds of stillbirth following a false-positive MSS (Figure 5). All triple-marker studies showed a positive association between false-positive MSS and increased odds of fetal demise, with 2 of 5 reaching statistical significance individually (pooled OR 2.46, 95% CI 1.94 to 3.12) [50, 52]. Only 2 of 3 of the double-marker studies showed increased odds of stillbirth, although none of these individual studies reached statistical significance (pooled OR 1.12, 95% CI 0.40 to 3.09). Of note, the triple-marker study by Naylor et al. also assessed risk of stillbirth but was not captured in the quantitative meta-analysis results because no cases of fetal demise were identified in either the false-positive MSS group of 50 or the control group of 100 pregnancies [51]. There was low heterogeneity amongst the double- and triple-marker screens, and only 14% of the heterogeneity amongst triple marker tests was attributed to random variation.

#### 3.3.3. Fetal Growth Restriction

**Figure 6:**
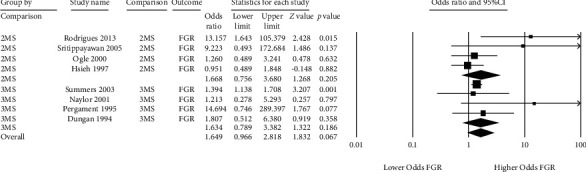
Forest plots of the relationship between false-positive MSS and fetal growth restriction (FGR), subgrouped by double- (2MS) and triple-marker (3MS) test type.

Eight studies assessed fetal growth restriction and met quality standards (Figure 6) [25, 26, 44–47, 51, 52]. Seven of the 8 studies showed evidence of increased odds of growth restriction amongst pregnancies with a false-positive MSS. Because of wide confidence intervals amongst most of the primary studies, the magnitude of this relationship was variable with only 2 of 8 individual studies reporting statistically significant results [46, 52]. The solitary study with an odds ratio of less than 1 reported that the true odds of fetal growth restriction in that cohort could range from 51% lower odds up to 85% higher odds with 95% certainty [44]. Overall, the pooled odds ratios were similar when comparing double- to triple-marker screens: 1.69 (95% CI 0.76 to 3.68) and 1.63 (95% CI 0.79 to 3.4), respectively. There was moderate heterogeneity amongst double-marker screens (*Q*‐df = 2.39, *I*^2^ 44.3%) and low heterogeneity amongst triple-marker screens (*Q*‐df < 0, *I*^2^ 0%).

#### 3.3.4. Preterm Birth

**Figure 7:**
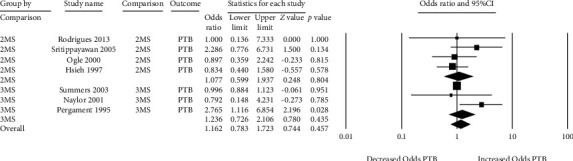
Forest plots of the relationship between false-positive MSS and preterm birth (PTB), subgrouped by double- (2MS) and triple-marker (3MS) test type.

There were 7 studies that measured the relationship with preterm birth (Figure 7) [25, 26, 44–46, 51, 52]. Both directionality and magnitude of odds ratios were variable. Amongst double-marker screens, 2 studies showed decreased odds of preterm birth, 1 study showed increased odds, and the fourth study was neutral with an odds ratio of 1 [26, 44–46]. The pooled odds ratio reflected this variability (odds ratio 1.07 with 95% CI 0.60 to 1.94) (Figure 7). Amongst triple-marker screens, one study showed decreased odds of preterm birth, one study showed increased odds, and the third study was neutral (odds ratio 0.996) [25, 51, 52]. It should be noted that the only study with a narrow confidence interval and a statistically significant result (*p* = 0.028) was the study by Pergament et al. which showed an increased risk of preterm birth with a false-positive triple-marker screen [25]. Overall, the pooled odds ratio amongst triple-marker studies was 1.24 (95% CI 0.73 to 2.11) (Figure 7). Heterogeneity was low for both double- and triple-marker screens (*I*^2^ 0 and *I*^2^ 25%, respectively).

### 3.4. Quality Assessment and Grade

Metaregression and sensitivity analyses were not possible for further evaluation of the influence of maternal age on outcomes due to small number of studies and limited maternal demographic information but were conducted to appraise the influence of the larger studies for each of the respective outcomes. Sensitivity analyses by inclusion of larger, poorer quality studies had limited impact on the main outcomes of preeclampsia (pooled OR 1.79 (1.05-3.03)) or fetal demise (pooled OR 2.22 (1.79-2.75) [49, 55]; there was also little influence on the pooled estimates for the heterogeneous and nonsignificant outcomes of fetal growth restriction (pooled OR 1.48 (0.94-2.32)) and preterm birth (pooled OR 1.07 (0.91, 1.27)) [24, 43, 49, 54, 55]. However, with increases in *I*^2^ (>25-50%) also noticed, the study heterogeneity proportionally worsened with the inclusion of the poorer quality studies.

Upon visual inspection of funnel plots, there appeared some evidence of publication bias regarding the outcomes of preeclampsia and fetal growth restriction (Supplementary Information Figures S2-S3), and the classic fail-safe Ns are 8 and 21, respectively; however, by Egger's tests, there was no statistical evidence of funnel plot asymmetry for preeclampsia (*p* = 0.15). But for fetal growth restriction, there was still significant publication bias and plot asymmetry noted (*p* = 0.02). There is no evidence of publication bias by visual inspection of funnel plots for stillbirth or preterm birth (Supplementary Information Figures S4-S5) or by Egger's tests: stillbirth (*p* = 0.45) and preterm birth (*p* = 0.27). An overview of the findings, including grading of evidence (comparing study results to baseline risks in the general population), is summarized in Table 2.

## 4. Discussion

This review was the first of its kind to address the relationship between false-positive, second-trimester, MSS using the screening cut-off for Down syndrome as a marker of risk for nongenetic complications of pregnancy. Traditionally, once aneuploidy and fetal structural anomalies are ruled out, patients with screen-positive MSS tests are reassured and discharged back into routine prenatal care; this meta-analysis suggests that this population may represent a higher-than-average risk group for developing placentally mediated complications later in pregnancy. Several groups including the World Health Organization have highlighted the urgent need for prioritisation of health policies and funding to reduce maternal and infant deaths at regional global levels given the growing burden of placentally mediated complications of pregnancy [27, 28]. Specifically for hypertensive disorders of pregnancy which comprise the leading cause of direct maternal deaths in developed countries, this review provides evidence that false-positive MSS tests in the second trimester may be early markers of disease later in pregnancy. The 28% increased odds of preeclampsia following false-positive MSS predicted by our study is significantly higher than the background population risk of 2.4-2.5% [37, 38, 56, 57]; improved awareness by healthcare providers of this relationship would allow for application of information already available from existing, routine prenatal genetic screening tests to enhance risk stratification of pregnancies for later complications without the need of additional tests or increased costs. The ability of earlier markers of preeclampsia to confer additional time and opportunities for prevention strategies could significantly reduce perinatal morbidity and warrant further study [20, 29].

We observed some evidence of a higher risk of stillbirth following a false-positive triple-marker MSS from this review: the odds of stillbirth following false-positive MSS was almost 2.5 times greater than that of screen-negative controls and well above the 0.5-3.5% background risk in the general population [36, 56]. Stillbirth is arguably one of the most devastating complications of pregnancy and can have risks for future pregnancy complications as well as long-lasting impacts on the mental health and well-being of affected families [58–62]. An enhanced understanding of the relationship between placental markers used for prenatal genetic screening and later pregnancy complications like stillbirth may offer chances for increased surveillance and/or other risk-reduction practices to improve outcomes. We found no significant association with preterm birth or fetal growth restriction following false-positive MSS for Down syndrome, likely reflecting the heterogeneity of potential causes of these conditions beyond placental function. A similar lack of association was seen amongst double-marker MSS, with pooled estimates that were much less robust and included wide confidence intervals which did not reach statistical significance. From our results, there appears to be improved precision of the odds ratio with the use of more serum markers, which is consistent with trends in improved prenatal detection of Down syndrome achieved when multiple additional serum markers are used [1, 7]. From these observations, one might infer that the same improvement would be seen with the use of quadruple-marker MSS in predicting placentally mediated complications of pregnancy; however, we were unable to evaluate this specific relationship given the lack of quality study studies evaluating quadruple-marker MSS.

While the random-effects model attenuates the impact of any individual study, it should be acknowledged that the study by Summers et al. carried the most weight for our analysis [52]. Had this study been eliminated, there would have been little effect on the association with preeclampsia given that all studies showed an increased risk in the setting of a false-positive MSS. There would also have been negligible impact on the odds of preterm birth given this study's finding of a “neutral” result. For stillbirth and fetal growth restriction, removal of this study would weaken the described association. The next largest study by Spencer et al. had to be excluded due to poor quality and high risk of bias, namely, the lack of adjustment for confounders [49]. In that study, there was no statistically significant association with fetal growth restriction or preterm birth, but this study did show a significant increase in the risk of stillbirth following a false-positive MSS [49]. Hence, the association between false-positive MSS and increased risk of stillbirth would have been even stronger had this study been included. However, the statistically insignificant results for growth restriction and preterm birth would have been attenuated even further.

This review offers practical insights for patient counseling as there are no other reviews published about second-trimester MSS using a screen-positive cut-off for comparison. A key strength of our review was in the ability to evaluate rare outcomes for which individual studies were often underpowered, particularly regarding stillbirth. There is also a perceived benefit of the relative “ease” with which healthcare providers may be able to implement these study findings clinically: use of already existing screen-positive cut-offs to distinguish between pregnancies at high versus low risk of complications is simpler than application of a complex algorithm comprised of random combinations of serum markers at random levels. The generalizability of this analysis was enhanced by inclusion of studies worldwide, and the restrictive inclusion criteria insured that heterogeneity between studies was minimized: there was also no restriction on dates of inclusion since there has been little if any change in the second-trimester MSS technology used since inception in the 1980s.

One limitation was the lack of quality studies comparing outcomes for the quadruple-marker MSS, which is currently the most widely utilized second-trimester MSS test. It is unclear why there are so few articles assessing MSS and the risk of adverse outcomes; however, there are some potential explanations for this phenomenon: (i) the initial studies evaluating nongenetic pregnancy complications after false-positive MSS reported no significant association because the odds ratios were less than 2, and many of these studies were also underpowered with lack of adjustment for potential confounders; (ii) by using arbitrary cut-offs of single analytes in the risk relationship with adverse pregnancy outcomes, it was impractical to implement the findings of earlier studies for individual patients; and (iii) many of the initial studies predated options for possible prevention and intervention strategies if a pregnancy was deemed to be at high risk for nongenetic complications [29, 63, 64]. Additionally, as many screening programs have transitioned to the first trimester or combined first- and second-trimester screening, there has been less focus on second trimester-only MSS (which remains the main publically funded testing option in our centre and in many other jurisdictions where patients present for care after the first trimester). Another limitation was incomplete information regarding maternal age, and other confounders precluded our ability to run a metaregression. Because this review was restricted to singleton pregnancies only, it is also unclear whether the association between false-positive MSS and the risk of adverse outcomes is maintained for twins or higher-order multiples. To date, most of the studies about multiples pertain to levels of individual serum markers; however, these do show consistency with our review and provide some evidence of a relationship between abnormal markers and increased risk of adverse pregnancy outcomes amongst multiples as well [65, 66]. Potential sources of bias of this review may include measurement (due to methods of ascertainment of cases), observer, and small study bias. While we were limited by a small number of investigators due to the nature of this original work done as part of course requirements, both authors have significant clinical and content expertise in obstetrics and prenatal screening, and JM is also an expert methodologist with valuable experience leading and conducting many systematic reviews.

The findings of this meta-analysis are of particular significance given current trends towards screening for fetal aneuploidy using cell-free fetal DNA in maternal circulation as opposed to the traditional serum marker-based screening tests [1, 2, 67]: if serum markers have the ability to enhance prediction of nongenetic pregnancy complications, this additional information may be lost with newer genetic screening technology and is worth consideration. Larger prospective studies that adequately control for confounders are still needed to validate these results, and future research is needed to evaluate how second-trimester MSS test results could be incorporated into prediction models and intervention strategies for the prevention of placentally mediated complications of pregnancy long-term. Additional studies are also required to synthesize potential risks of adverse pregnancy outcomes following false-positive first-trimester and integrated first- and second-trimester screening tests. At a minimum, this review suggests that the current clinical practice of simply reassuring patients with positive MSS once genetic diagnoses are ruled out and returning them to routine prenatal care should be reevaluated.

## 5. Conclusions

There is evidence that false-positive, second-trimester MSS is a marker of increased risk of preeclampsia and stillbirth later in pregnancy. However, this evidence is limited by the small overall number and sample sizes of individual studies. Additional large-scale studies that are sufficiently powered for rare outcomes and adequately adjusted for confounders are still needed to obtain more reliable estimates of risk and prediction modelling as well as evaluation of potential interventions to mitigate these disease risks. It will also be important to study the utility of first-trimester screening tests as even earlier markers of placentally mediated complications given they have become the more popular modality of serum-based aneuploidy screening for women presenting for care in the first trimester. Obstetrical care providers should be aware of this potential association when counseling patients: even after aneuploidy has been ruled out, these pregnancies may still remain at higher-than-average risk of complications. Aneuploidy screening programs which are transitioning towards cell-free fetal DNA-based testing may lose the additional information offered by serum marker-based testing, and this should be considered when planning health policies for preventing maternal and neonatal morbidity.

## Figures and Tables

**Table 1:** Characteristics of eligible studies.

Study	Design	Setting	Study period	Exclusions	Cases	Controls	Test & cut-off	Outcomes
Dungan (1994) [47]	Prospective cohort (matched)	Tennessee, USA (regional medical centre screening database)	Jan 1990 to Dec 1991	Multiples, age ≥ 35 y	False-pos DS screen (*n* = 99)	Screen Neg (matched by age, race, parity, and bleeding) (*n* = 99)	Triple marker (15-20 wk GA) ≥1 : 270 at midgest	IUFD, BWt < 5%ile, PET, major anomalies
Pergament (1995) [25]	Retrospective cohort (matched)	Illinois, USA (large teaching hospital)	Jul 1990 to Jun 1991	Multiples, age ≥ 35 y	False-pos DS screen (*n* = 58)	Screen Neg (matched by age, race, sample date, DM) (*n* = 116)	Triple marker (15-20 wk GA) ≥1 : 250 at midgest	PTB, PPROM, abruption, PET, BWt < 10%ile, IUFD, APOs
Milunsky (1996) [48]	Retrospective cohort (matched)	Massachusetts, USA (university teaching hospital)	1992 to 1994	Multiples, DM	Abn lytes plus screen Pos for DS (*n* = 38)^∗^	Normal lytes plus screen Neg for DS (matched by age, GA, and race) (*n* = 76)	Abnormal analyte levels +/- triple marker (15-24 wk GA) ≥1 : 270 at midgest	LBW, IUFD, NND, anomalies, DS
Chapman (1997) [43]	Retrospective cohort	Alabama, USA (genetic database at university teaching hospital)	Aug 1988 to Feb 1995	Age < 30 y, anomalies, abn amnio	False-pos DS screen plus normal amnio (*n* = 246)	Screen Neg plus normal amnio (*n* = 889)	Double marker (15-20 wk GA) ≥1 : 190 at midgest	IUFD, PTB, BWt < 10%ile
Hsieh (1997) [44]	Retrospective cohort ^∗^*adjusted analysis*	Taipei, Taiwan (university hospital)	Jan 1994 to Feb 1996	Age < 35 y	False-pos DS screen (*n* = 311)	Screen Neg (*n* = 4982)	Double marker (14-22 wk GA) ≥1 : 270 at midgest	PTB, postdates, BWt < 10%ile, LGA, macrosomia, PET, PPH, PROM, abruption, anomalies, poly, oligo, IUFD
Ogle (2000) [45]	Retrospective cohort (matched)	London, UK (university teaching hospital)	22 mo period	Multiples, preg dating not done by us	False-pos DS screen (*n* = 272)	Screen Neg (matched by age and GA of testing) (*n* = 272)	Double marker (15-18 wk GA) ≥1 : 250 at term	IUFD, PTB, PET, IUGR, APOs
Spencer (2000) [49]	Retrospective cohort	UK (tertiary hospital prenatal screening program)	1995 to 1998	Multiples, anomalies, abn amnio, preg losses < 24 wk GA	Cases = abn outcome (*n* = 3728)	Controls = normal outcome (*n* = 26524)	Triple marker (15-19 wk GA) >1 : 251 at midgest	PTB, SGA, IUGR, IUFD
Antsaklis (2001) [50]	Retrospective cohort (matched)	Athens, Greece (large teaching hospital)	Jan 1991 to Dec 1998	Multiples, age > 35 y	False-pos DS screen (*n* = 454)	Screen Neg (matched by age & GA at testing) (*n* = 912)	Triple marker (16-20 wk GA) ≥1 : 250 at midgest	Fetal death (total and by etiology)
Naylor (2001) [51]	Prospective cohort (matched)	California, USA (university teaching hospital)	Aug 1995 (start)	Non-Hispanics, multiples, HTN, DM, PMHx, anomalies	False-pos DS screen plus normal amnio (*n* = 50)	Screen Neg, matched (*n* = 100)	Triple marker (15-20 wk GA) ≥1 : 190 at midgest	PET, PTB, BWt < 10%ile, IUFD, APOs
Summers (2003) [52]	Retrospective cohort (matched)	Canada (provincial genetic database)	Oct 1995 to Sep 1998	Multiples, DM, +NTD screens, anomalies	False-pos DS screen (*n* = 11549)	Screen Neg (matched by age, ethnicity, test site, sample date) (*n* = 11549)	Triple marker (15-20 wk GA) ≥1 : 385 at term	PIH, PET, APH, PROM, SGA, PTB, oligo, poly, IUFD, APOs
Sritippayawan (2005) [26]	Retrospective cohort (matched)	Bangkok, Thailand (university teaching hospital)	Mar 1998 to Aug 2002	Multiples, DM, anomalies, aneuploidy, missing info, POBHx or FamHx, +NTD or unknown screen	False-pos DS screen (*n* = 165)	Screen Neg (matched by age, parity, and date of testing) (*n* = 165)	Double marker (14-21 wk GA) ≥1 : 270 at midgest	PTB, LBW, SGA, PET, previa, IUFD, APOs
Xia (2006) [53]	Retrospective cohort	Shanghai, China (university teaching hospital)	Apr 2002 to Mar 2005	Multiples	False-pos DS screen (*n* = 233)	Screen Neg (*n* = 4322)	Triple marker (13-21 wk GA) ≥1 : 384 at term	IUGR, BGI, NBI, OSB, IUFD, distress, APOs
Weintraub (2012) [54]	Retrospective cohort	Negev, Israel (university teaching hospital)	ᴓ	Abnormal levels of individual analytes	False-pos DS screen (normal lytes & amnio) (*n* = 123)	Screen Neg (*n* = 482)	Triple marker (15-20 wk GA) ≥1 : 380 at term	Abn lytes, amnio, GDM, poly, IUGR, PROM, PTB, postdates, IOL, augmentation, breech, FTP, distress, mec, mode, GA, BWt, gender, Apgars, LGA, AGA, SGA, cardiac anomalies, PPH
Rodrigues (2013) [46]	Retrospective cohort (matched)	Lisbon, Portugal (hospital database)	Mar 2003 to Aug 2007	Multiples	False-pos T1 (*n* = 57 + 60) or T2 double marker screen (*n* = 62)	Screen Neg (matched by age, GA at testing, and type of screen) (*n* = 62)	PN screening cohort of 4422 (T1 integrated or T1 serum integrated or T2 double marker) vs controls	PTB, IUGR, HDP
Godbole (2015)	Retrospective cohort	Pune, India (tertiary care private hospital)	2 years	Abnormal chx	“High risk” screen (*n* = 189)	“Low risk” screen (*n* = 157)	Triple marker (*n* = 136 + 77) vs T1 double marker (*n* = 53 + 80)	PTB, LBW, oligo, CS, PIH, NICU admission, resp distress
Yazdani (2015) [55]	Retrospective cohort	Babol, Iran (university teaching hospital)	Mar 2012 to 2013	Chronic HTN, DM, renal dis, heart dis, thal, thyroid dis, multiples, family hx, pos amnio	False-pos DS screen (*n* = 80)	Screen Neg (*n* = 151)	Quad marker (14-18 wk GA)	Composite APO, anemia, mode of del, infertility, UTI, GDM, GHTN, PET, PTL, IUGR, poly, oligo, PROM, skin dis, abruption

Legend: abn = abnormal; AGA = appropriate for gestational age; amnio = amniocentesis; APH = antepartum hemorrhage; APO(s) = abnormal pregnancy outcome(s); BGI = basal ganglia injury; BWt = birthweight; chx = chromosome; CS = cesarean section; del = delivery; dis = disease; DM = diabetes mellitus; DS = Down syndrome; FamHx = family medical history; GA = gestational age; GDM = gestational diabetes; GHTN = gestational hypertension; HDP = hypertensive disorders of pregnancy; HTN = hypertension; IOL = induction of labour; IUFD = intrauterine fetal demise or stillbirth; IUGR = intrauterine growth restriction; LWB = low birthweight; LGA = large for gestational age; lytes = analytes; midgest = midgestation of pregnancy or “midpregnancy”; NBI = neonatal brain injury; Neg = negative; NND = neonatal death; oligo = oligohydramnios; OSB = open spina bifida; PET = preeclampsia; PIH = pregnancy-induced hypertension; PMHx = past medical history; POBHx = past obstetrical history; poly = polyhydramnios; pos = positive; preg = pregnancy; PPH = postpartum hemorrhage; PROM = premature rupture of membranes; PTB = preterm birth; PTL = preterm labour; resp = respiratory; SGA = small for gestational age; T1 = first-trimester; T2 = second-trimester; thal = thalassemia; US = ultrasound; UTI = urinary tract infection; +NTD screens = positive screen for neural tube defect; %ile = percentile; ?screen = unknown or missing screening results.

**Table 2:** Summary of findings and quality of evidence (GRADE), overall and by a triple-marker maternal serum screening test.

Outcomes	Estimated risk (baseline risk in population)^a^	Total number of participants (# of studies)	Pooled OR (95% CI)	Number of participants in TMS studies (# of studies)	Pooled OR, by TMS (95% CI)	Quality of evidence (GRADE)^b^	Comments
Preeclampsia (PET)	2.6-3.5%	*n* = 24494 (6 studies)	1.28 (1.09-1.51)	*n* = 23620 (4 studies)	1.28 (1.09-1.51)	Moderate	Consistency of findings; low heterogeneity (low-mod for TMS); low-risk publication bias
Stillbirth (IUFD)	0.5-0.6% (high-income countries) vs. 1.5-3.5% (low-income countries)	*n* = 31117 (8 studies)	1.95 (0.96-3.96)	*n* = 24950 (5 studies)	2.46 (1.94-3.12)	Moderate	Stronger relationship and narrower CIs for TMS tests vs. pooled overall; low heterogeneity; low-risk publication bias
Fetal growth restriction (FGR)	3-10%^c^	*n* = 29911 (8 studies)	1.65 (0.97-2.82)	*n* = 23620 (4 studies)	1.63 (0.79-3.38)	Low	Significant publication bias; inconsistency of results
Preterm birth (PTB)	8-10%	*n* = 29713 (7 studies)	1.16 (0.78-1.72)	*n* = 23422 (3 studies)	1.24 (0.73-2.11)	Low	Significant heterogeneity & inconsistency of results between studies

Legend: TMS = triple-marker maternal serum screening tests; OR = odds ratio; CI = confidence interval. ^a^See References ^∗∗^[36–38, 56–62]. ^b^See Reference [42]. ^c^Fetal growth restriction using <10^th^ percentile as a cut-off correlates with baseline population risk ~10%. However, within the literature, there is a range of reported risks (between 3 and 10%) based on the different growth percentile cut-offs used.

## Data Availability

The data used to support the findings of this study are available from the corresponding author upon request.

## Supplementary Materials

Supplementary Materials Table S1: sample search strategy for PubMed database. Figure S1: standardized data collection sheet. Figure S2: funnel plot for publication bias with trim and fill to evaluate preeclampsia. Figure S3: funnel plot for publication bias with trim and fill to evaluate stillbirth. Figure S4: funnel plot for publication bias with trim and fill to evaluate fetal growth restriction. Figure S5: funnel plot for publication bias with trim and fill to evaluate preterm birth. Table S2: PRISMA checklist for systematic reviews.
