# Supplementary material for: False-Positive Maternal Serum Screens in the Second Trimester as Markers of Placentally Mediated Complications Later in Pregnancy: A Systematic Review and Meta-Analysis
Source: Dis Markers. 2021 Jun 4;2021:5566234. doi: 10.1155/2021/5566234 (PMC8295507; doi:10.1155/2021/5566234)
Supplement: Supplementary Materials — Table S1: sample search strategy for PubMed database. Figure S1: standardized data collection sheet. Figure S2: funnel plot for publication bias with trim and fill to evaluate preeclampsia. Figure S3: funnel plot for publication bias with trim and fill to evaluate stillbirth. Figure S4: funnel plot for publication bias with trim and fill to evaluate fetal growth restriction. Figure S5: funnel plot for publication bias with trim and fill to evaluate preterm birth. Table S2: PRISMA checklist for systematic reviews. [file 5566234.f1.zip › Figure S1.doc]

| Methods | Study Design:  Dates:  “Cases”:  “Controls”: | Location:  Setting  Other:  Follow-Up: |
| --- | --- | --- |
| Participants | Sample Size:  Baseline Characteristics:  Identification process? | Eligibility:  Exclusion Criteria: |
| Intervention | Type of MSS testing:  Timing of test:  Screening cutoff: | Pregnancy dating?:  Amniocentesis?:  How were test results identified?: |
| Outcome(s) | How were outcomes identified?: |  |
| Notes |  |  |
