# Supplementary material for: False-Positive Maternal Serum Screens in the Second Trimester as Markers of Placentally Mediated Complications Later in Pregnancy: A Systematic Review and Meta-Analysis
Source: Dis Markers. 2021 Jun 4;2021:5566234. doi: 10.1155/2021/5566234 (PMC8295507; doi:10.1155/2021/5566234)
Supplement: Supplementary Materials — Table S1: sample search strategy for PubMed database. Figure S1: standardized data collection sheet. Figure S2: funnel plot for publication bias with trim and fill to evaluate preeclampsia. Figure S3: funnel plot for publication bias with trim and fill to evaluate stillbirth. Figure S4: funnel plot for publication bias with trim and fill to evaluate fetal growth restriction. Figure S5: funnel plot for publication bias with trim and fill to evaluate preterm birth. Table S2: PRISMA checklist for systematic reviews. [file 5566234.f1.zip › Figures S2 S3 S4 S5.doc]

Figure S2: Funnel plot for publication bias with trim and fill to evaluate preeclampsia.

Figure S3: Funnel plot for publication bias with trim and fill to evaluate stillbirth.

Figure S4: Funnel plot for publication bias with trim and fill to evaluate fetal growth restriction.

Figure S5: Funnel plot for publication bias with trim and fill to evaluate preterm birth.
