# Supplementary material for: False-Positive Maternal Serum Screens in the Second Trimester as Markers of Placentally Mediated Complications Later in Pregnancy: A Systematic Review and Meta-Analysis
Source: Dis Markers. 2021 Jun 4;2021:5566234. doi: 10.1155/2021/5566234 (PMC8295507; doi:10.1155/2021/5566234)
Supplement: Supplementary Materials — Table S1: sample search strategy for PubMed database. Figure S1: standardized data collection sheet. Figure S2: funnel plot for publication bias with trim and fill to evaluate preeclampsia. Figure S3: funnel plot for publication bias with trim and fill to evaluate stillbirth. Figure S4: funnel plot for publication bias with trim and fill to evaluate fetal growth restriction. Figure S5: funnel plot for publication bias with trim and fill to evaluate preterm birth. Table S2: PRISMA checklist for systematic reviews. [file 5566234.f1.zip › Table S1.doc]

| Search | Query | Items found |
| --- | --- | --- |
| #37 | Search #36 AND English[Language] | 2166 |
| #36 | Search #19 AND #29 AND #35 | 2386 |
| #35 | Search #30 or #31 or #32 or #33 or #34 | 746764 |
| #34 | Search (pregnancy[title/abstract] OR pregnant[title/abstract] OR obstetric*[title/abstract] OR maternal[title/abstract] OR fetal[title/abstract] OR fetus[title/abstract] OR medical[title/abstract]) AND outcome*[title/abstract] | 284748 |
| #33 | Search (pregnancy[title/abstract] OR pregnant[title/abstract] OR obstetric*[title/abstract] OR maternal[title/abstract] OR fetal[title/abstract] OR fetus[title/abstract] OR medical[title/abstract]) AND adverse[title/abstract] | 72909 |
| #32 | Search (pregnancy[title/abstract] OR pregnant[title/abstract] OR obstetric*[title/abstract] OR maternal[title/abstract] OR fetal[title/abstract] or fetus[title/abstract] or medical[title/abstract]) AND complication*[title/abstract] | 142171 |
| #31 | Search "pregnancy complication*"[title/abstract] | 670 |
| #30 | Search "Pregnancy Complications"[Mesh] | 416168 |
| #29 | Search #20 or #21 or #22 or #23 or #24 or #25 or #26 or #27 or #28 | 960941 |
| #28 | Search abnormal*[title/abstract] | 686940 |
| #27 | Search unexplained[title/abstract] | 34217 |
| #26 | Search discordan*[title/abstract] | 28308 |
| #25 | Search diagnostic uncertain*[title/abstract] | 1324 |
| #24 | Search misdiagnos*[title/abstract] | 31134 |
| #23 | Search diagnostic error*[title/abstract] | 3809 |
| #22 | Search false-positive[title/abstract] | 46254 |
| #21 | Search false[title/abstract] | 113105 |
| #20 | Search "Diagnostic Errors"[Mesh] | 114209 |
| #19 | Search #1 or #2 or #3 or #4 or #5 or #6 or #7 or #8 or #9 or #10 or #11 or #12 or #13 or #14 or #15 or #16 or #17 or #18 | 26675 |
| #18 | Search ((prenatal test*[title/abstract]) or (prenatal screen*[title/abstract])) AND (biomarker*[title/abstract] or serum[title/abstract] or noninvasive[title/abstract] or blood[title/abstract] or analyte*[title/abstract]) | 1879 |
| #17 | Search "Prenatal diagnosis"[Mesh] AND ("Biomarkers"[Mesh] or "blood"[sh] or noninvasive[title/abstract] or serum[title/abstract] or analyte*[title/abstract]) | 10937 |
| #16 | Search aneuploidy screen*[title/abstract] | 609 |
| #15 | Search quad test*[title/abstract] | 6 |
| #14 | Search quad screen*[title/abstract] | 39 |
| #13 | Search quadruple test*[title/abstract] | 60 |
| #12 | Search quadruple screen*[title/abstract] | 22 |
| #11 | Search triple marker[title/abstract] | 147 |
| #10 | Search triple test*[title/abstract] | 415 |
| #9 | Search maternal serum test*[title/abstract] | 45 |
| #8 | Search maternal serum screen*[title/abstract] | 632 |
| #7 | Search "maternal serum screening tests"[Mesh] | 447 |
| #6 | Search "Down Syndrome/diagnosis"[Mesh] | 6063 |
| #5 | Search "Down Syndrome/blood"[Mesh] | 1500 |
| #4 | Search "Trisomy/diagnosis"[Mesh] | 1212 |
| #3 | Search "Trisomy/blood"[Mesh] | 4 |
| #2 | Search "Chromosome Disorders/diagnosis"[Mesh] | 14113 |
| #1 | Search "Chromosome Disorders/blood"[Mesh] | 2572 |
